# Transparency in ovarian cancer clinical trial results: ClinicalTrials.gov versus PubMed, Embase and Google scholar

**DOI:** 10.1186/s13048-018-0404-1

**Published:** 2018-04-10

**Authors:** Anna Roberto, Silvia Radrezza, Paola Mosconi

**Affiliations:** Laboratory for medical research and consumer involvement, IRCCS Istituto di Ricerche Farmacologiche Mario Negri, Via G. La Masa 19, 20156 Milan, Italy

**Keywords:** Transparency, Clinical trials, ClinicalTrials.gov, Ovarian cancer, Publication of results

## Abstract

**Background:**

In recent years the question of the lack of transparency in clinical research has been debated by clinicians, researchers, citizens and their representatives, authors and publishers. This is particularly important for infrequent cancers such as ovarian cancer, where treatment still gives disappointing results in the majority of cases. Our aim was to assess the availability to the public of results in ClinicalTrials.gov, and the frequency of non-publication of results in ClinicalTrials.gov and in PubMed, Embase and Google Scholar. We collected all trials on ovarian cancer identified as “completed status” in the ClinicalTrials.gov registry on 17 January 2017. We checked the availability of the results in ClinicalTrials.gov and systematically identified published manuscripts on results.

**Results:**

Out of 2725 trials on ovarian cancer identified, 752 were classified as “completed status”. In those closed between 2008 and 2015, excluding phase I, the frequency of results in ClinicalTrials.gov was 35%. Of the 752 completed studies the frequency of published results in PubMed, Embase or Google Scholar ranged from 57.9% to 69.7% in the last years.

**Conclusions:**

These findings show a lack of transparency and credibility of research. Citizens or patients’ representatives, with the medical community, should continuously support initiatives to improve the publication and dissemination of clinical study results.

## Background

The lack of transparency of clinical research results has long been on the agenda of clinicians [1], researchers, citizens and their representatives [2, 3], authors and publishers [4, 5]. The AllTrials petition on the transparency of clinical data is attracting widespread interest [4], and the “Restoring Invisible and Abandoned Trials” initiative is making public the confidential results about missing and abandoned trials [6]. “Better reporting of better research” is the motto shared by citizens and patients [7], and also underlined recently by members of the European Parliament [8].

To facilitate access to clinical trial information, in 1997 the database ClinicalTrials.gov was developed through the US Food and Drug Administration Modernization Act. This is the world’s largest clinical trial registry, public and accessible to citizens [9, 10]. In 2005, the “International Committee of Medical Journal Editors” implemented a policy requiring prospective registration of clinical trials for publication in its member journals [11]. In 2007, the US Food and Drug Administration Amendments Act [FDAAA] required trial results to be reported in ClinicalTrials.gov, within one year from completion and regardless of whether the results had been published [12]. Finally, in 2008, the ClinicalTrials.gov registry expanded its database to include a section reporting basic summary results [13, 14]. Nevertheless, compliance with the reporting of results continued to be lacking, probably because some statutory requirements were ambiguous.

In September 2016, the US Department of Health and Human Services published a final rule to increase the accountability of all clinical research stakeholders by expansion of minimum data sets about legal requirements under FDAAA for trial registration and submission of results. The final rule became effective on 18 January 2017 through those responsible had until 18 April 2017 to become familiar and compliant with it. At the same time, the Nation Institute of Health issued a complementary final policy [15].

Ovarian cancer can be considered a rare disease in comparison with other major oncological killers, even if it is the fifth most common cancer among women. About 65,000 women are diagnosed every year in Europe, and 42,000 die each year [16]. No early diagnosis is available and the results of treatment are still poor for the majority of cases. It is therefore essential that any results of clinical research, positive or negative, be published to permit broad discussion of the different strategies for care.

The aims of this study were to evaluate the availability of basic summary results of registered clinical trials in ovarian cancer in ClinicalTrials.gov, and the frequency of non-publication of results in ClinicalTrials.gov and in PubMed, Embase or Google Scholar.

## Methods

From January to September 2017, we examined all completed clinical trials on ovarian cancer registered in ClinicalTrials.gov in terms of the frequency of results published in PubMed, Embase and Google Scholar.

### ClinicalTrials.gov database

Using the “advanced search” function of ClinicalTrials.gov we searched for trials on ovarian cancer. Only those listed as “completed status”, indicating that the trial was ended normally, were included. All these reports were downloaded as an XML data set (12 January 2017). For each one we considered the following: starting date, study design (observational or interventional), type of study (diagnostic, therapeutic, supportive care, prevention and screening, health services research), country, multicentric (yes or no), funding source (profit or not-for-profit), number of primary outcomes, nature of the primary outcomes, enrolment of participants, and sample size. These variables collected in the ClinicalTrials.gov registration database are described in the Data Element Definitions document issued by the National Library of Medicine [17]. Trials not on ovarian cancer or closed after 2015 were excluded.

### PubMed database

For each trial in this analysis, we used the link within ClinicalTrials.gov in order to identify the published article. If this was not possible, we systematically searched for the publication in PubMed, Embase and Google Scholar through the following keywords: trial unique registration number, trial title, principal investigator’s name, and treatment. We considered a trial as “published” when a peer-reviewed publication was found, either online or in print, including any data. We then confirmed the publication identity by comparing inclusion and exclusion criteria, trial arms, type of outcomes, treatment and comparator, sample size and primary investigator names in the registration record. For each article the year of publication and the journal, the primary and secondary outcomes, sample size and impact factor (IF) were collected. We examined the correspondence between the journal publication and trial registered, as reported in ClinicalTrials.gov. The outcome correspondence analysis was conducted by IF less or more than 10 and by profit or not-for-profit. Finally, we calculated the mean time to publication of the results.

### Basic summary results section in ClinicalTrials.gov

As the section reporting summary results has been available in ClinicalTrials.gov since 2008, we verified whether it had been completed on a subsample of the trials that ended between 2008 and 2015. Phase I trials were not considered as their results are not required to be published in ClinicalTrials.gov [12]. The variables collected in the ClinicalTrials.gov registration database are described in the Basic Results Data Element Definitions document issued by the National Library of Medicine [18]. Generally this section reports descriptive statistics on the characteristics of patients enrolled, and the results for the primary and secondary outcomes.

### Statistical analyses

For continuous variables, means, medians and standard deviations were obtained. For categorical variables, proportions were obtained. All descriptive analyses were done using SAS, version 9.4.

## Results

**Fig. 1:**
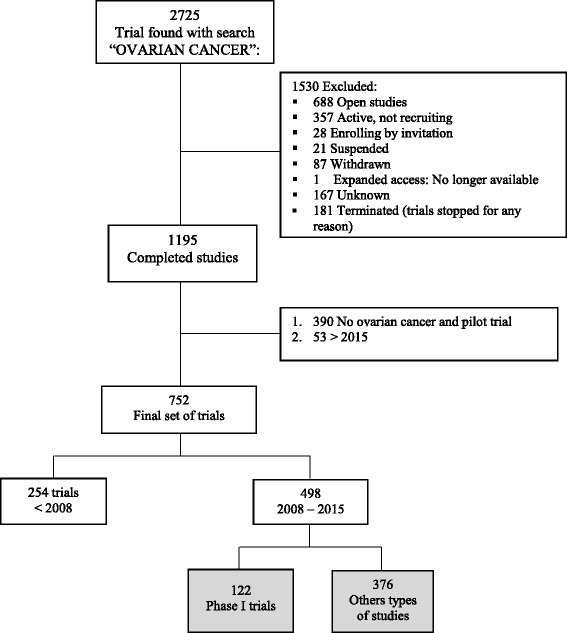
Flow-chart describing the selection of ovarian cancer trials and the availability of results

As described in the flow-chart (Fig. 1), out of 2725 studies retrieved by the search through ClinicalTrials.gov we identified 1195 with “completed status”. The final set, related to ovarian cancer and closed in 2015, comprised 752 completed studies. To examine this database we considered the following groups: 254 completed before 2008; 122 completed Phase I trials closed between 2008 and 2015; 376 other completed studies closed between 2008 and 2015.

Table 1 illustrates the main features of completed studies. Most were Phase II, multicentre trials, conducted in America, for treatment purposes. Safety and efficacy were the most common endpoints, and major were not-for-profit. There was a large amount of missing data for the endpoint classification and center. The mean duration was 4.5 years.

**Table 1:** Main features of the trials reported in ClinicalTrials.gov

ClinicalTrials.gov studies	All (752)		< 2008 (254)		2008–2015 (498)	
	N	%	N	%	N	%
Study phase						
0	3	0.4	–	–	3	0.6
I	186	24.7	67	26.4	119	23.9
I-II	54	7.2	21	8.3	33	6.6
II	313	41.6	114	44.9	199	40.0
II-III	6	0.8	2	0.8	4	0.8
III	67	8.9	21	8.3	46	9.2
IV	7	0.9	2	0.8	5	1.0
Observational	71	9.4	18	7.1	53	10.6
Missing	45	6.0	9	3.5	36	7.2
Center						
Multicenter	393	52.3	106	41.7	287	57.6
Single-center	208	27.7	76	29.9	132	26.5
Missing	151	20.1	72	28.4	79	15.9
Country^a^						
America	489	65.0	195	76.8	294	59.0
Europe	114	15.2	25	9.8	89	17.9
International	99	13.1	17	6.7	82	16.5
Asia	21	2.8	4	1.6	17	3.4
Oceania	3	0.4	–	–	3	0.6
Study purpose^a^						
Treatment	612	81.4	213	83.9	399	80.1
Prevention and screening	20	2.7	6	2.4	14	2.8
Diagnostic	15	2.0	2	0.8	13	2.6
Supportive care	29	3.9	12	4.7	17	3.4
Health services research	5	0.7	1	0.4	4	0.8
Other	34	4.5	4	1.6	30	6.0
Missing	37	5.0	16	6.3	21	4.2
Endpoint classification						
Efficacy	168	22.3	67	26.4	101	20.3
Safety	80	10.6	27	10.6	53	10.6
Safety and efficacy	256	34.0	68	26.8	188	37.8
Other	106	14.1	18	7.1	88	17.7
Missing	142	19.0	74	29.1	68	13.7
Sponsor						
Not-for-profit	463	61.6	189	74.4	274	55.0
Profit	289	38.4	65	25.6	224	45.0
Study duration (years)						
Mean (SD)	4.48 (2.8)		4.12 (2.9)		4.64 (2.7)	
Range	1–24		1–24		1–21	
Number of subjects						
< 50	382	51.6	142	37.2	240	62.8
≥ 50	359	48.5	105	29.3	254	70.8

^a^Some discrepancies in the total are due to missing data; less than 5% of data are missing

The mean time to journal publication was 2.5 years (SD 1.8, range 1–11) only for reports published after the end of the trials (data not shown).

Table 2 compares the primary and secondary outcomes reported in published articles and in the trials registered in ClinicalTrials.gov. The frequency of publication of results in the ClinicalTrials.gov section among “other studies” completed between 2008 and 2015 is nearly 35%. For the primary outcome, 51.8% of all trials corresponded, but 37.8% were not declared in ClinicalTrials.gov. The figures for secondary outcome were 39.3% of all trials. In this case, the percentage of secondary outcomes not declared in ClinicalTrials.gov reached 44.5%.

**Table 2:** Trials published and correspondence between outcomes in the publication and ClinicalTrials.gov

	N	%	N	%	N	%	N	%
Results Reported in	All (752)		< 2008 (254)		2008-2015^a^ (498)			
					Phase I (122)		Other studies types (376)	
ClinicalTrials.gov	NA		NA		NA		130	34.6
Journal publication	483	64.2	147	57.9	74	60.7	262	69.7
Outcome correspondence	All (483)		< 2008 (147)		Phase I (74)		Other studies types (262)	
Primary Outcome								
Corresponded	249	51.8	40	27.0	37	50.7	172	66.2
Not corresponded	50	10.4	13	8.8	7	9.6	30	11.5
Not declared in ClinicalTrials.gov	182	37.8	95	64.2	29	39.7	58	22.3
Secondary Outcome								
Corresponded	157	39.3	24	19.1	27	41.5	106	50.7
Not corresponded	65	16.2	14	11.1	7	10.8	44	21.1
Not declared in ClinicalTrials.gov	178	44.5	88	69.8	31	47.7	59	28.2

*NA*, Not applicable

^a^See the number reported in the highlighted boxes in Fig. 1

A total of 94 reports of trials completed between 2008 and 2015 did not report results either in ClinicalTrials.gov or in a publication available in PubMed, Embase or Google Scholar (Table 3).

**Table 3:** Results in ClinicalTrials.gov and journal publication for trials ended between 2008 and 2015 (N 376)

	ClinicalTrials.gov	
Journal publication	Yes - *N (%)*	No - *N (%)*
Yes - *N (%)*	110 (29.3)	152 (40.4)
No - *N (%)*	20 (5.3)	94 (25.0)
Total	130 (34.6)	246 (65.4)

Comparison of the information reported in the published articles and in the results’ section of ClinicalTrials.gov (Table 4) shows several discrepancies between what was declared and what was actually published. One hundred and five different journals were found, with an average IF of 10.3 (range 1.5–72.4). For 10 of them no IF was declared. On primary outcomes the correspondence between journal publication and ClinicalTrials.gov trials is similar in both groups (Table 4). Outcome correspondence was less common in the journals with IF≥10. In the high IF journals it was more frequent not to declare primary outcome in ClinicalTrials.gov.

**Table 4:** Correspondence between outcomes in publications and ClinicalTrials.gov, based on Impact Factor

Outcome correspondence based on Impact Factor	Impact factor ≥ 10 (18^a^)		Impact factor < 10 (75^a^)		Total (93)	
	N	%	N	%	N	%
Primary Outcome						
Corresponded	19	73.1	62	79.5	81	77.9
Not corresponded	2	7.7	11	14.1	13	12.5
Not declared in ClinicalTrials.gov	5	19.2	5	6.4	10	9.6
Secondary Outcome						
Corresponded	11	57.9	36	54.6	47	55.3
Not corresponded	5	26.3	16	24.2	21	24.7
Not declared in ClinicalTrials.gov	3	15.8	14	21.2	17	20.0

^a^Number of journals with IF less than 10 or more according to each subgroup. The same journal could have published several articles

The correspondence for secondary outcomes was similar in both groups. These results suggest that the quality of outcome reported was not significantly influenced by the IF.

We retrieved a total of 463 not-for-profit trials and 289 for profit. Only studies published between 2008 and 2015 were analyzed. The main features such as trial phase, center, purpose, etc., were described and compared (Table 5).

**Table 5:** Main features of not-for-profit and profit trials ended between 2008 and 2015

ClinicalTrials.gov studies	Not-for-profit 2008–2015 (274)		Profit 2008–2015 (224)	
	N	%	N	%
Study phase				
0	3	16.5	–	–
I	46	19.0	73	33.2
I-II	18	7.4	15	6.8
II	103	42.6	96	43.6
II-III	–	–	4	1.8
III	29	12.0	17	7.7
IV	3	1.2	2	0.9
Observational	40	16.5	13	5.9
Missing	32		4	
Center				
Multicenter	125	58.7	162	78.6
Single-center	88	41.3	44	21.4
Missing	61		18	
Country				
America	178	66.4	116	53.5
Europe	64	23.9	25	11.5
International	16	6.0	66	30.4
Asia	8	3.0	9	4.2
Oceania	2	0.8	1	0.5
Study purpose				
Treatment	196	76.0	203	92.7
Prevention and screening	10	3.9	4	1.8
Diagnostic	8	3.1	5	2.3
Supportive care	15	5.8	2	0.9
Health services research	3	1.2	1	0.5
Other	26	10.1	4	1.8
Missing	16			
Endpoint classification				
Efficacy	71	32.0	30	14.4
Safety	18	8.1	35	16.8
Safety and efficacy	78	35.1	110	52.9
Other	55	24.8	33	15.9
Missing	52		16	
Study duration (years) - Mean (SD)	5.27 (3.00)		3.74 (2.17)	
Number of subjects				
< 50	128	46.7	112	50.0
> =50	143	52.2	111	49.5

As for the IF, we verified the outcome correspondence between journals and ClinicalTrials.gov not-for-profit and profit trials separately. The correspondence of primary outcomes was similar in both samples with a total correspondence between two sources of 76.2%. For the secondary outcome, however, for profit trials corresponded more closely, with 64.2% vs. 43.2% respectively (Table 6).

**Table 6:** Correspondence between outcomes in publications and ClinicalTrials.gov among “not-for-profit” vs “profit” studies

Outcome correspondence	Not-for-profit (52)		Profit (58)	
	N	%	N	%
Primary Outcome^a^				
Corresponded	39	76.5	44	75.9
Not corresponded	7	13.7	7	12.1
Not declared in ClinicalTrials.gov	5	9.8	7	12.1
Secondary Outcome^a^				
Corresponded	16	43.2	34	64.2
Not corresponded	12	32.4	11	20.8
Not declared in ClinicalTrials.gov	9	24.3	8	15.1

^a^Some discrepancies in the total are due to missing data

## Discussion

Several papers indicate a worrisome percentage of clinical trials whose results are not available for clinicians, researchers and citizens or patients [2, 19–23]. To our knowledge this topic is still less deepened in ovarian cancer field. Some publications are consistent with our results showing there is still a general need for transparency in publication of findings. Guo and colleagues reported that 69% of a sample of 35 clinical trials on endometriosis were unpublished [19]. As regards the links between ClinicalTrials.gov and PubMed, Huser and Cimino [20] reported that the majority of 8907 trials (72%] had no structured trial-article link available, and 55% of so-called “silent” trials had no linked results article or even basic summary results. Such under-reporting can contribute to biased evidence, with serious consequences for clinical practice, research and, in the end, for patients [21, 24]. Pranic and Marusic [25] report a similar result about 81 eligible trials found in ClinicalTrials.gov between 2009 and 2012. In this sample, the secondary outcomes were left out for 17% at the initial registration and for 19.7% at the last.

Basing on our analysis aimed on ovarian cancer, very high percentages of completed ovarian cancer clinical trials - about a quarter - do not see the light either in the specific results section of ClinicalTrials.gov or in medical journals.

Also important are the inconsistencies between the trials registered and the published primary and secondary outcomes. These inconsistencies are also present in the ClinicalTrials.gov results section. From the viewpoint of citizens or patients a complete results section in a public registry such as ClinicalTrials.gov should offer an easy way to find complete information about all registered clinical trials. On the single level, this could facilitate decision-making on new treatments or even new clinical trials [1, 26]. At the community level, for example for patients’ associations, the results could help in identifying uncertainties or answering questions about the effects of treatments and discussing research priorities. From the researchers’ point of view the lack of access to all trial results contributes to publication bias [2], to misuse of systematic reviews, meta-analyses and clinical guidelines and, finally, reduces the possibility of designing innovative research. Even more important, from the clinicians’ point of view this lack of information on results can lead to an unfair doctor-patient relationship, where advice on care and treatment is not in line with the latest knowledge.

Researchers and sponsors have a great responsibility to report results and data related to their clinical research fully, as recently underlined by the new European Medicines Agency regulation on trial publication [27].

Nevertheless, it is not easy to investigate the cause of this marked lack of transparency as many factors can contribute, such as career, market and sponsors, or conflict of interest. Probably until a few years ago some researchers did not even consider the lack of transparency an important issue that could help improve the quality of research and reduce waste. Moreover there was no public opinion movement, such as the AllTrials campaign [4], which could lead to greater transparency. And yet ensuring greater transparency would only need a little effort on the part of researchers, who should be anxious to register publicly their trials and make the results public as required by FDAAA [13]. It also defined criteria and a checklist of mandatory registration data or documents, to register and then to publish results [28]. To guarantee a standard method for reporting results, there are also international guidelines such as CONSORT 2010 (Consolidated Standards of Reporting Results) [29] and ICH E3 (International Conference on Harmonisation of Technical Requirements for Registration of Pharmaceuticals for Human Use) [30].

In addition, to improve the clarity and boost the spread of information, data sharing is encouraged and is considered a new “gold standard” by all scientific stakeholders; several initiatives or projects are planned to increase it. Researchers can, through specific platforms, have access to anonymized patients’ data from several clinical trials, and other documentation to conduct further research [31]. As regards the homogenization of procedures to plan and conduct a clinical trial, the European Community has drawn up the New Regulation 536/2014. Its first aim is to improve the transparency of the data collected by all European trials and to make them public. The Regulation also defines the standards to assure the safety and quality of research, fostering collaboration among Member States [32].

Recently, the editors of peer-reviewed journals have become more willing to publish negative trials results and to check the consistency of information to be published with that reported in registers such as ClinicalTrials.gov [25].

Finally, citizens, patients and their associations can all do something to obtain greater transparency in research: for example, associations and public funders can decide to finance only trials which expressly state they will adhere to the rules of transparency.

As ovarian cancer is so infrequent, disclosure of study results is an ethical duty from the moment patients are enrolled, and this should be fully stated in the informed consent form. The latest version of the Declaration of Helsinki exhorts researchers, authors, sponsors, editors and publishers to report and disseminate positive or negative results of clinical research [33]. What patient would be willing to participate in a clinical trial if the data collected will not be useful to anyone - neither the scientific community nor other patients? Associations of citizens or patients could be also more forceful in demanding more transparency in reporting results, and offer their support only to researchers or multicenter groups that guarantee full disclosure.

The sample of trials that did not report the results either in a journal or in the results section in ClinicalTrials.gov amounted to about 30% of those on recurrent platinum-resistant ovarian cancer, and about 30% with advanced ovarian cancer. Only a few trials involved newly-diagnosed women or those in the first line of treatment. Nearly half the trials were Phase II, and one-tenth were observational.

In our sample not only was the results section often missing, but single items required during the registration in ClinicalTrials.gov were often lacking too: for example, the nature of the study, or the endpoint classification. On the other hand, it is worth noting the limited amount of data missing among all the items required now for registration in the clinical trials whose results were reported both in ClinicalTrials.gov and in a journal. This shows that a high level of data reporting is possible with little extra effort.

The inconsistencies between the trial registered and the published results on primary and secondary outcomes are not to be overlooked; these inconsistencies are also present in the ClinicalTrials.gov results section but also in journals with IF of 10 or more. In fact, a quality analysis based on the IF it does not show any appreciable differences between the two groups. These results are in line with a previous cross-sectional analysis [34] of 96 clinical trials. These trials were published in 2010–2011 in PubMed, in high-impact journals (IF≥10) and the results were registered and reported in ClinicalTrials.gov; nearly all had at least one discrepancy in the cohort, intervention, primary endpoints or results reported in the two sites. In addition, Smith et al. [35] reported disparity for 79% of the registry-publication pairs especially about primary outcomes.

Results that we retrieved were similar for the analysis among not-for-profit and for-profit trials. This implies, that efforts to improve the quality and transparency of data published are still needed especially for secondary outcomes.

At the end of a trial, the analysis should include publication of the results on the basis of the primary outcome on which the trial was designed, and also the sample size. Not disclosing the primary outcome, but only the secondary one, can have a detrimental impact in the scientific community, who do not receive the right information, and on patients who participated in the not properly designed trial. Moreover, future studies can be damaged too as other researchers can may design new trials on the basis of previous uncorrected results. Finally, it is unethical to ask a patient informed consent for to take part in a trial with a defined endpoint, and then not to make the result public.

This analysis has some limitations. Misclassification of clinical trials might have influenced the data collection, as well as the lack of updating by investigators. In addition, the analysis was restricted to the period between 2008 and 2015 and some journal articles might have been missed, considering the time needed for peer review before publication. As concern strengths, this paper adds evidence to the discussion about the lack of transparency of clinical research, showing that the problem is far from solved.

## Conclusions

Researchers, clinicians and citizens or patients’ representatives need to focus on transparency as an important ethical issue and continuously support initiatives to improve the publication and dissemination of clinical findings. The publication of protocols and statistical analysis plans could be useful to ensure transparency through the investigators themselves should be more compliant or explain discrepancies [36]. In addition, publishers and editors should pay close attention to ensure that information reported in ClinicalTrials.gov is consistent with the information reported in papers submitted.

## Abbreviations

FDAAA: Food and Drug Administration Amendments Act
IF: Impact Factor
IRCCS: Istituto di Ricovero e Cura a Carattere Scientifico
SAS: Statistical Analysis Software
XML: Extensible Markup Language
